# CT semi-quantitative score used as risk factor for hyponatremia in patients with COVID-19: a cross-sectional study

**DOI:** 10.3389/fendo.2024.1342204

**Published:** 2024-06-14

**Authors:** Baofeng Wu, Ru Li, Jinxuan Hao, Yijie Qi, Botao Liu, Hongxia Wei, Zhe Li, Yi Zhang, Yunfeng Liu

**Affiliations:** ^1^ Department of Endocrinology, First Hospital of Shanxi Medical University, Taiyuan, China; ^2^ First Clinical Medical College, Shanxi Medical University, Taiyuan, China; ^3^ Department of Medical Imaging, Shanxi Medical University, Taiyuan, China; ^4^ Department of Pharmacology, Shanxi Medical University, Taiyuan, China

**Keywords:** COVID-19, SARS-CoV-2, hyponatremia, computed tomography, free triiodothyronine, pneumonia

## Abstract

**Purpose:**

Chest computed tomography (CT) is used to determine the severity of COVID-19 pneumonia, and pneumonia is associated with hyponatremia. This study aims to explore the predictive value of the semi-quantitative CT visual score for hyponatremia in patients with COVID-19 to provide a reference for clinical practice.

**Methods:**

In this cross-sectional study, 343 patients with RT-PCR confirmed COVID-19, all patients underwent CT, and the severity of lung lesions was scored by radiologists using the semi-quantitative CT visual score. The risk factors of hyponatremia in COVID-19 patients were analyzed and combined with laboratory tests. The thyroid function changes caused by SARS-CoV-2 infection and their interaction with hyponatremia were also analyzed.

**Results:**

In patients with SARS-CoV-2 infection, the total severity score (TSS) of hyponatremia was higher [M(range), 3.5(2.5–5.5) vs 3.0(2.0–4.5) scores, *P*=0.001], implying that patients with hyponatremia had more severe lung lesions. The risk factors of hyponatremia in the multivariate regression model included age, vomiting, neutrophils, platelet, and total severity score. SARS-CoV-2 infection impacted thyroid function, and patients with hyponatremia showed a lower free triiodothyronine (3.1 ± 0.9 vs 3.7 ± 0.9, *P*=0.001) and thyroid stimulating hormone level [1.4(0.8–2.4) vs 2.2(1.2–3.4), *P*=0.038].

**Conclusion:**

Semi-quantitative CT score can be used as a risk factor for hyponatremia in patients with COVID-19. There is a weak positive correlation between serum sodium and free triiodothyronine in patients with SARS-CoV-2 infection.

## 1 Introduction

Hyponatremia is a common electrolyte disorder in hospitalized patients, often associated with poor prognosis (1). Severe hyponatremia may cause complications, such as cerebral edema, seizures, and coma. Patients with community-acquired pneumonia (CAP) are more likely to have hyponatremia (Na^+^<135mmol/L), and hyponatremia is associated with more extended hospital stays, increased hospital costs, and increased mortality (2). The link between COVID-19 and hyponatremia is well known, and multiple studies have described the prevalence of hyponatremia in COVID-19 patients ranging from 20% to 35% (3). COVID-19 causes hyponatremia in patients likely to have the following several aspects: the first is due to the SARS-CoV-2 infection increases interleukin 6 (IL-6) (4), and IL-6 can cross the blood-brain barrier and directly stimulate the supraoptic and paraventricular nuclei cause the syndrome of inappropriate antidiuresis (SIAD) (5); Secondly, SARS-CoV-2 enters host cells through the angiotensin converting enzyme 2(ACE2), and its binding to ACE2 will down-regulate the activity of ACE2, causing an imbalance between ACE and ACE2, destroying the renin-angiotensin-aldosterone system (RAAS), and leading to the accumulation of angiotensin II (6). Animal studies have found that local application of various components of RAS to the paraventricular nucleus and supraventricular nucleus of the hypothalamus can trigger the release of hypothalamus antidiuretic hormone (ADH), which may also be the cause of hyponatremia in COVID-19 patients (7); Finally, electrolyte disturbances can also be caused by inappropriate use of diuretics and hypotonic fluids in patients with excessive fluid load for treatment.

Most patients with SARS-CoV-2 infection present with pneumonia, and the most common symptoms include fever, cough, dyspnea, and sore throat. Chest CT is an essential and helpful technique for diagnosing and evaluating lung diseases, including pneumonia. CT can detect the signs of pulmonary involvement of COVID-19 and can be used for highly sensitive diagnosis earlier than the reverse transcription-polymerase chain reaction (RT-PCR) test results, which is helpful to quickly and accurately determine the severity of the disease to carry out reasonable management and treatment of patients (8, 9). Many chest CT scoring systems have been developed to assess the severity of lung involvement, and the TSS is widely used (10, 11).

This study explored the association between semi-quantitative CT visual score and endocrine-related factors in patients with SARS-CoV-2 infection and hyponatremia, providing evidence for the vital role of CT score in pneumonia diagnosis, disease severity stratification, and prognosis analysis.

## 2 Materials and methods

### 2.1 Study design

This study was a cross-sectional study. Patients admitted to the First Hospital of Shanxi Medical University and diagnosed with COVID-19 from January 1 to January 31, 2023, were included. The study was approved by the Ethics Committee of the First Hospital of Shanxi Medical University (Approval number:2018K002). The patients/participants provided written informed consent to participate in this study.

Inclusion criteria:

1. SARS-CoV-2 infection was positive by RT-PCR 2. Chest CT showed definite pulmonary infection 3. Age ≥18 years old

Exclusion criteria:

1. Patients who were missing CT imaging data and laboratory indicators 2. Patients with hypernatremia 3. Patients were readmitted due to COVID-19 and transferred patients

**Figure 1:**
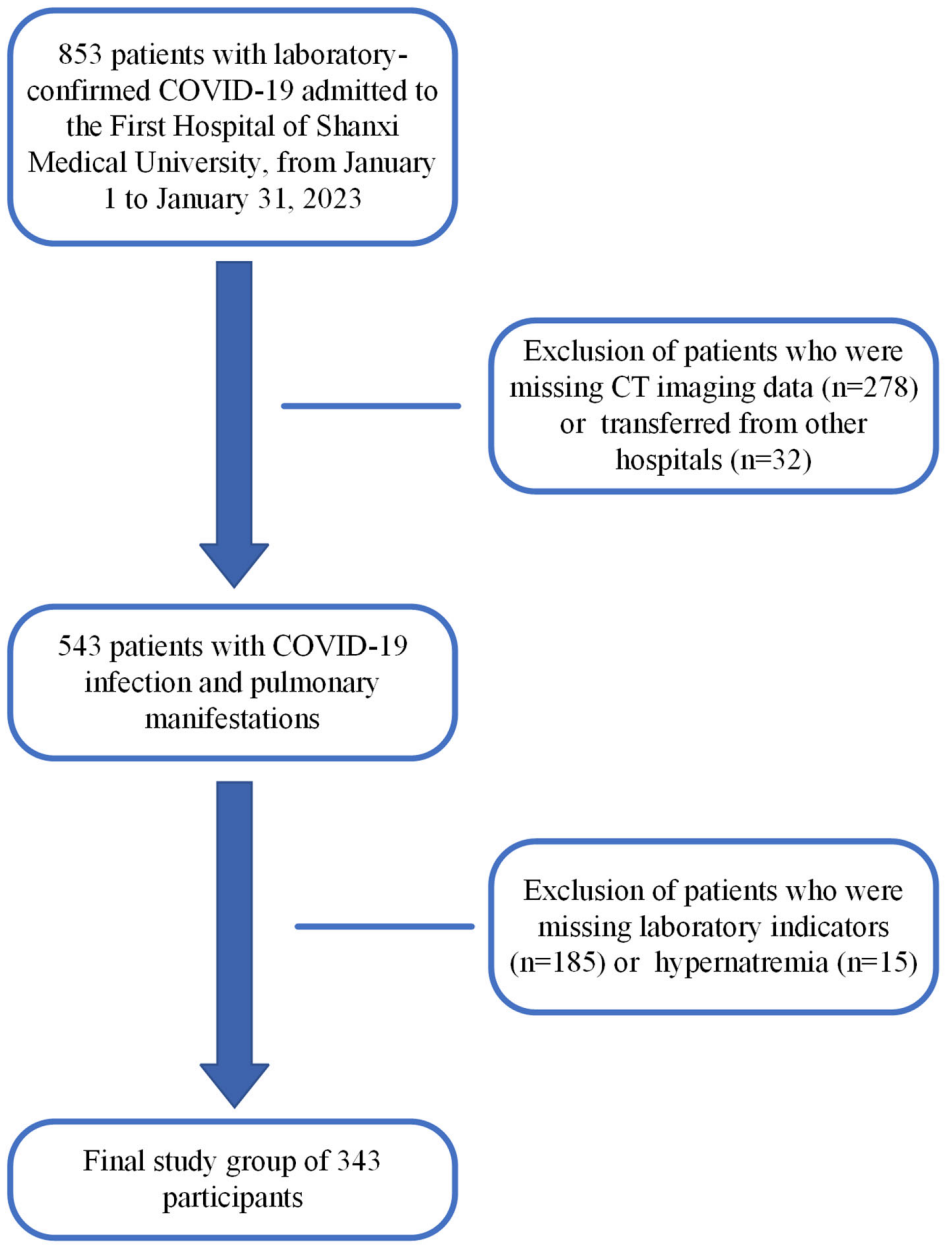
Flowchart of the study design.

According to inclusion and exclusion criteria, 343 patients were included in the final study (**Figure 1**). The purpose of this study is to explore the CT semi-quantitative score of COVID-19 patients with hyponatremia prediction effect. Therefore, patients included in the study must demonstrate the presence of SARS-CoV-2 infection and complete data on the underlying laboratory tests and examinations, and those who did not meet these criteria were excluded. Second, given the rarity of hypernatremia in COVID-19 patients (prevalence of 3.7% to 7%) (12, 13), we also excluded patients with hypernatremia because only 15 patients had hypernatremia in this study, which could not meet the statistical requirements.

### 2.2 Definition of covariates

The variables studied included age, sex, vital signs, symptoms, comorbidities, medication at admission, and laboratory parameters. The clinical symptoms we collected included fever, shortness of breath, cough/expectoration, muscle soreness, disturbance of consciousness, poor appetite, vomiting, and diarrhea. Comorbidities collected included diabetes, hypertension, coronary heart disease, cerebral infarction, thyroid dysfunction, and pulmonary disease. Medications on admission included diuretics, ACEI/ARBs, and glucocorticoids. Laboratory indicators included blood cell analysis, liver and kidney function indicators, coagulation indicators, electrolytes, inflammatory indicators, and other indicators. Blood cell analysis is measured by instrumental method (CAL8000), the determination of liver and kidney function by adopting the method of rate method and bromocresol green method, electrolytic determination with ion selective electrode (indirect method), coagulation function is measured by coagulation method and immunoturbidimetric method (ACL TOP 550), BNP and PCT are measured by microparticle chemiluminescence method and thyroid function is measured by electrochemiluminescence method (COBAS 6000).

Hyponatremia was defined as serum sodium less than 135 mmol/L, measured mainly by the indirect ion-selective electrode (ISE) method. Patients were further classified as having mild, moderate, or severe hyponatremia if their serum sodium levels were 130 to <135 mmol/L, 125 to <130 mmol/L, and <125 mmol/L, respectively.

### 2.3 CT image acquisition and interpretation

All patients underwent a chest CT scan on admission. CT image data were obtained from one of four CT scanners (GE Lightspeed VCT 64, GE HealthCare, American; Somatom Force, Siemens Healthineers, Germany; IQon Spectral CT, Philips Healthcare, The Netherlands; NeuViz 128 CT, Neusoftmedical, China). The CT scan was performed with the patient supine and at the end of inspiration without administering intravenous contrast material. The scanning range was from the apex to the base of the lung. According to the international recommendations and other studies (14, 15), the parameters used were tube voltage (120kV) and tube current (60–100 mA), which were set by the automatic exposure control system (iDose) program, and the image quality was customized according to the needs of low dose patients. Thoracic VCAR pulmonary function analysis software (AW VolumeShare 7, GE company, American) was used to analyze the image data. The 0.625 mm slice thickness image at the end of deep inspiration was post-processed, and the threshold limit (-1024 to -200 HU) and automatic segmentation technology were used. The heart, trachea, rib, and other lung tissues were segmented to obtain a three-dimensional lung tissue model.

### 2.4 Semiquantitative CT visual score

In this study, we used the TSS to analyze chest CT findings in hospitalized patients. TSS is a digital scoring system based on visual evaluation that analyzes the range of lesions in CT images. Two radiologists with years of experience in imaging diagnosis performed scoring. To more clearly express mild and moderate hyponatremia CT score difference, our lung lesions (ground-glass opacity, consolidation, GGO + consolidation) to the following classification: 0:0%; 1:1–10%; 2: 11–20%; 3:21–30%; 4:31–40%; 5:41–50%; 6: >50%. According to these percentages, 0,1,2,3,4,5 and 6 points are given, respectively. The final TSS was the total score of the left and right lungs (range 0–12).

### 2.5 Statistical analysis

The conformity of the data to a normal distribution was evaluated using skewness, kurtosis tests, and histogram plots. Normally distributed continuous variables are presented as mean and standard deviation (± SD); a Student *t*-test was used. Non-normalized variables were presented as medians with interquartile ranges, and a Mann-Whitney *U* test was used. Categorical variables are described as the number (percentage), and Chi-square or Fisher’s exact tests were used. Multivariate analysis was carried out using Logistic Regression (Forward Selection: Likelihood Ratio) to determine the significant risk factors of hyponatremia. The analysis of variance (ANOVA) was used in normal distribution variables, and the Kruskal Wallis test was used in non-normal distribution variables to compare the hyponatremia group (mild/moderate/severe) and the difference between normonatremia group. The data was entered and analyzed using the IBM SPSS 27 system (SPSS Inc., Chicago, IL, USA). A *P*-value ≤5% was taken for statistical significance.

## 3 Results

### 3.1 General characteristics of COVID-19 patients

A total of 343 eligible patients were included in the study, 58.6% male. The mean age of the patients was 74.5 ± 13.1 years, and 89.5% were older than 60 years. Cough/expectoration (74.9%), poor appetite (66.2%), and shortness of breath (44.6%) were the most common clinical symptoms observed. Hypertension and diabetes were the most common comorbidities, accounting for 49.0% and 25.9%, respectively. Of the 343 study patients, 43.4% had hyponatremia, whereas 56.6% had normonatremia (**Table 1**). Among the patients with hyponatremia, the prevalence of mild, moderate, and severe hyponatremia was 53.7%, 16.8%, and 29.5%, respectively (**Supplementary Table S1**).

**Table 1:** Demographic and clinical characteristics of the patients.

	Normonatremia (*n*=194)	Hyponatremia (*n*=149)	*P*-value
Demographic characteristics			
Age (years)	72.5 (65.0–82.8)	80.0 (70.0–86.5)	**<0.001**
Male, *n* (%)	110.0 (56.7)	91.0 (61.1)	0.415
Body mass index (kg/m^2^)	23.9 (21.1–26.8)	24.2 (21.3–26.7)	0.769
Vital signs			
Body temperature (°C)	36.5 (36.3–36.8)	36.6 (36.3–37.0)	0.087
Pulse (Times/min)	80.0 (76.0–90.0)	80.0 (73.5–90.5)	0.430
SBP (mmHg)	130.0 (118.0–139.8)	132.0 (118.0–144.0)	0.325
DBP (mmHg)	76.0 (70.0–82.0)	76.0 (69.0–82.5)	0.839
Symptoms			
Fever, *n* (%)	28.0 (14.4)	31.0 (20.8)	0.121
Shortness of breath, *n* (%)	93.0 (47.9)	60.0 (40.3)	0.157
Cough/Expectoration, *n* (%)	153.0 (78.9)	104.0 (69.8)	0.055
Muscle soreness, *n* (%)	26.0 (13.4)	14.0 (9.4)	0.252
Disturbance of consciousness, *n* (%)	12.0 (6.2)	16.0 (10.7)	0.127
Poor appetite, *n* (%)	124.0 (63.9)	103.0 (69.1)	0.312
Vomiting, *n* (%)	11.0 (5.7)	21.0 (14.1)	**0.008**
Diarrhea, *n* (%)	6.0 (3.1)	6.0 (4.0)	0.641
Comorbidities			
Diabetes, *n* (%)	45.0 (23.2)	44.0 (29.5)	0.185
Hypertension, *n* (%)	97.0 (50.0)	71.0 (47.7)	0.666
Coronary heart disease, *n* (%)	31.0 (16.0)	16.0 (10.7)	0.162
Cerebral infarction, *n* (%)	28.0 (14.4)	22.0 (14.8)	0.931
Thyroid dysfunction, *n* (%)	7.0 (3.6)	4.0 (2.7)	0.863
Pulmonary disease, *n* (%)	5.0 (2.6)	9.0 (6.0)	0.108
Medication at admission			
Diuretics, *n* (%)	6.0 (3.1)	15.0 (10.1)	**0.008**
ACEI/ARBs, *n* (%)	15.0 (7.7)	17.0 (11.4)	0.245
Glucocorticoids, *n* (%)	15.0 (7.7)	8.0 (5.4)	0.386
Laboratory tests			
Leukocyte (×10^9^/L)	5.7 (4.1–7.8)	6.2 (4.8–9.9)	**0.008**
Hemoglobin (g/L)	137.0 (124.0–148.0)	134.0 (121.5–144.0)	0.186
Platelets (×10^9^/L)	194.0 (142.0–246.0)	173.0 (124.5–229.5)	**0.016**
Lymphocytes (×10^9^/L)	1.0 (0.7–1.4)	0.8 (0.5–1.1)	**<0.001**
Neutrophils (×10^9^/L)	3.8 (2.4–6.5)	4.7 (3.3–7.9)	**<0.001**
NLR	3.8 (2.5–6.8)	7.3 (3.5–12.9)	**<0.001**
Blood glucose (mmol/L)	6.7 (5.8–8.8)	7.0 (6.2–9.2)	0.058
ALT (U/L)	20.5 (13.0–35.3)	27.0 (18.0–38.5)	**0.022**
AST (U/L)	27.0 (21.0–41.0)	37.0 (24.0–57.0)	**<0.001**
Albumin (g/L)	35.8 (33.0–39.3)	34.5 (31.8–38.1)	**0.028**
BUN (mmol/L)	5.3 (4.0–7.3)	5.5 (4.1–7.9)	0.558
SCr (μmol/L)	67.0 (57.0–81.6)	71.0 (56.0–88.5)	0.466
eGFR (mL/min/1.73m^2^)	91.5 (75.7–100.1)	85.2 (68.9–95.0)	**0.004**
Potassium (mmol/L)	3.9 (3.6–4.3)	3.9 (3.5–4.2)	0.197
Chlorine (mmol/L)	102.7 (100.4–104.8)	93.8 (87.2–98.1)	**<0.001**
PT (s)	13.6 (12.8–14.4)	13.4 (12.7–14.3)	0.392
APTT (s)	31.6 (29.3–33.7)	32.3 (29.9–35.9)	**0.035**
FDP (ug/ml)	4.7 (1.8–163.5)	4.4 (1.9–133.5)	0.504
D dimer (mg/L)	2.2 (0.3–4.5)	1.8 (0.3–4.4)	0.639
PCT (ng/ml)	0.26 (0.16–0.35)	0.29 (0.20–0.45)	**0.011**
BNP (ng/L)	52.6 (26.8–122.8)	109.0 (48.0–270.9)	**<0.001**
hs-cTnT (pg/ml)	9.5 (4.4–19.6)	13.5 (7.3–35.5)	**<0.001**
CT assessment			
TSS (scores)	3.0 (2.0–4.5)	3.5 (2.5–5.5)	**0.001**

Values are expressed as mean (± standard deviation), median (interquartile range), or number (percentage). Serum creatinine (SCr) measurements were used to calculate the estimated Glomerular Filtration Rate (eGFR) by using the 2021 Chronic Kidney Disease Epidemiology Collaboration (2021 CKD-EPI) Creatinine equation (16). SBP, systolic blood pressure; DBP, diastolic blood pressure; ACEI/ARB, angiotensin-converting enzyme inhibitor/angiotensin receptor blocker; NLR, neutrophil to lymphocyte ratio; ALT, Alanine aminotransferase; AST, aspartate aminotransferase; BUN, blood urea nitrogen; Cr, creatinine; eGFR, estimated glomerular filtration rate; PT, prothrombin time; APTT, activated partial thromboplastin time; FDP, fibrinogen degradation products; PCT, procalcitonin; BNP, brain natriuretic peptide; hs-cTnT, high-sensitivity cardiac troponin T; TSS, total severity score.

A P-value <0.05 was considered statistically significant, shown in bold.

Patients in the hyponatremia group were significantly older [M(range), 80.0(70.0–86.5) vs 72.5(65.0–82.8) years old, *P* < 0.001] than those in the normonatremia group. Vomiting (14.1% vs 5.7%, *P*=0.008) and diuretic use (10.1% vs 3.1%, *P*=0.008) in the hyponatremia group were significantly different from those in normonatremia group. The remaining measures of vital signs, symptoms, coexisting conditions, and out-of-hospital medication use did not differ significantly between the two groups (**Table 1**).

### 3.2 Laboratory findings and TSS

Among the laboratory indicators in **Table 1**, the median (IQR) findings of complete blood count (white blood cell/platelet/hemoglobin/neutrophil), renal function indexes (BUN, SCr), ALT, potassium, coagulation indicators (PT, APTT, and FDP), and BNP were within normal limits. Compared with the normonatremia group, the white blood cells and neutrophils in the hyponatremia group increased, while the platelets and lymphocytes decreased, and the difference was statistically significant (all *P*< 0.05, **Table 1**). Compared with the normonatremia group, the median eGFR and chlorine in the hyponatremia group were lower than the lower limit of normal, and the difference was statistically significant (all *P*< 0.05, **Table 1**). The median blood glucose, D dimer, and PCT in the hyponatremia group were increased, which were higher than the upper limit of normal, and the difference was statistically significant (all *P*< 0.05, **Table 1**).

**Figure 2:**
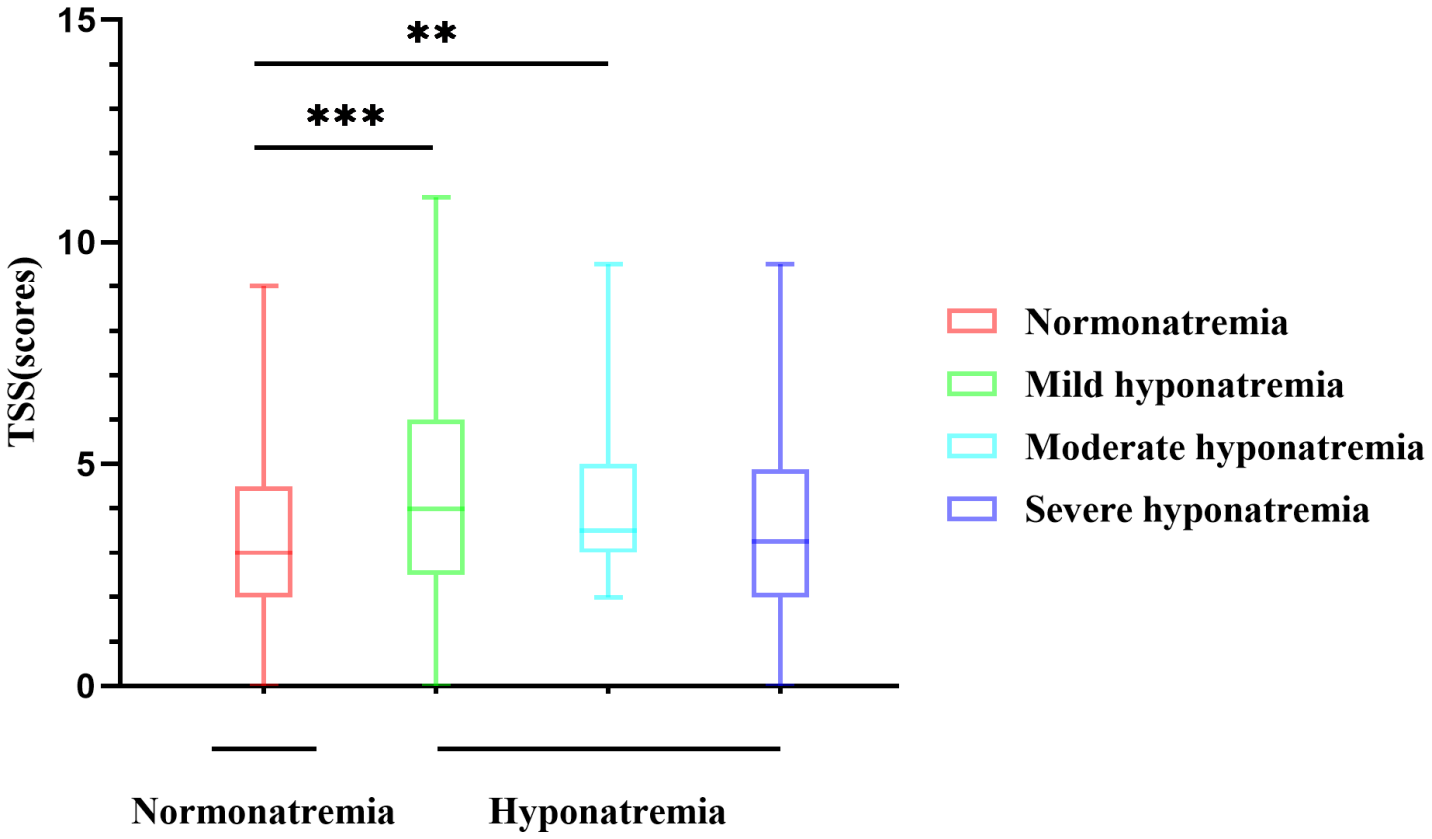
Differences in CT scores of different degrees of hyponatremia. The difference between the normonatremia and mild hyponatremia groups, and the normonatremia and moderate hyponatremia groups were significant. **P<0.01, ***P<0.001.

Excellent agreement was achieved between the two radiologists in the assessment of lung lesions, with an average measurement intraclass correlation coefficient (ICC) of 0.953 (95% CI, 0.942–0.962; *P* < 0.001) (**Supplementary Table S2**). Thus indicating a high reliability of the semi-quantitative method, in the following analysis, the average of the CT scores of the two radiologists was selected instead of using the scores of radiologist 1 or radiologist 2. For TSS, the hyponatremia group showed higher scores than the normonatremia group [M(range), 3.5(2.5–5.5) vs 3.0(2.0–4.5) scores, *P*=0.001] (**Table 1**), indicating more severe lung lesions. The hyponatremia was further divided into mild, moderate, and severe groups, and the difference in CT scores between different degrees of hyponatremia group and normonatremia group was analyzed. The results showed that the difference between the normonatremia and mild hyponatremia groups (*P*=0.001) and the normonatremia and moderate hyponatremia groups (*P*=0.023) were significant (**Figure 2**).

### 3.3 Risk factors for hyponatremia in patients with SARS-CoV-2 infection

Univariate and multivariate logistic regression analyses were performed to explore the risk factors of hyponatremia in patients with SARS-CoV-2 infection, combined with the statistics of the difference between the normonatremia group and the hyponatremia group. By univariate logistic regression analysis, statistically significant risk factors for hyponatremia included age, vomiting, diuretics, platelets, lymphocytes, neutrophils, NLR, eGFR, APTT, BNP, PCT, and TSS, as shown in **Table 2**.

**Table 2:** Logistic regression analysis to predict the indicators of hyponatremia in patients with COVID-19.

	Univariate model		Multivariate model	
	OR (95% CI)	*P*-value	OR (95% CI)	*P*-value
Demographic characteristics				
Age (years)	1.036 (1.017–1.056)	**<0.001**	1.039 (1.018–1.061)	**<0.001**
**Symptoms**				
Vomiting	2.729 (1.272–5.858)	**0.010**	2.920 (1.233–6.913)	**0.015**
Diarrhea	1.315 (0.415–4.162)	0.642		
Medication at admission				
Diuretics	3.507 (1.327–9.274)	**0.011**		
ACEI/ARBs	1.537 (0.741–3.189)	0.248		
Glucocorticoids	0.677 (0.279–1.642)	0.388		
Laboratory parameters				
Platelets (×10^9^/L)	0.997 (0.994–0.999)	**0.016**	0.995 (0.991–0.998)	**0.002**
Lymphocytes (×10^9^/L)	0.494 (0.329–0.749)	**<0.001**		
Neutrophils (×10^9^/L)	1.038 (1.013–1.171)	**0.001**	1.167 (1.079–1.263)	**<0.001**
NLR	1.053 (1.024–1.083)	**<0.001**		
ALT (mmol/L)	1.000 (0.996–1.004)	0.848		
AST (mmol/L)	1.002 (0.999–1.005)	0.196		
Albumin	0.968 (0.928–1.009)	0.124		
eGFR (mL/min/1.73m^2^)	0.990 (0.981–1.000)	**0.039**		
APTT (s)	1.036 (0.999–1.075)	0.058		
PCT (ng/ml)	1.062 (1.003–1.125)	**0.040**		
BNP (ng/L)	1.001 (1.000–1.002)	**0.007**		
hs-cTnT (pg/ml)	1.001 (0.999–1.003)	0.226		
CT assessment				
TSS (scores)	1.220 (1.094–1.361)	**<0.001**	1.203 (1.069–1.354)	**0.002**

OR, odds ratio; CI, confidence intervals; ACEI/ARB, angiotensin-converting enzyme inhibitor/angiotensin receptor blocker; AST, aspartate aminotransferase; eGFR, estimated glomerular filtration rate; APTT, activated partial thromboplastin time; PCT, procalcitonin; BNP, brain natriuretic peptide; TSS, total severity score.

A P-value <0.05 was considered statistically significant, shown in bold.

Based on our clinical observations, fluid loss due to diarrhea/vomiting and medications such as diuretics, ACEI/ARBs, or glucocorticoids can cause electrolyte disturbances. After excluding the variables with higher degree of collinearity, multivariate logistic regression analysis was performed, and the results showed that (**Table 2**) age (OR=1.039, 95%CI 1.018–1.061, *P*<0.001), vomiting (OR=2.920, 95%CI 1.233–6.913, *P*=0.015), neutrophil count (OR=1.167, 95%CI 1.079–1.263, *P*<0.001), TSS score (OR=1.203, 95%CI 1.069–1.354, *P*=0.002), and platelet count (OR=0.995, 95%CI 0.991–0.998, *P*=0.002) were independent risk factors for hyponatremia in COVID-19 patients.

### 3.4 The thyroid function between normonatremia group and hyponatremia group

To explore the role of endocrine-related factors in developing hyponatremia in COVID-19 patients, we performed a subgroup analysis of 104 patients with available thyroid function data. The results showed (**Table 3**) that the levels of free triiodothyronine (FT3), free thyroxine (FT4), and thyroid stimulating hormone (TSH) were within the normal range. However, the levels of FT3, FT4, and TSH in the hyponatremia group were lower than those in the normonatremia group, and the difference in FT3 (3.1 ± 0.9 vs 3.7 ± 0.9, *P*=0.001) and TSH [M (range), 1.4 (0.8–2.4) vs 2.2 (1.2–3.4) uIU/ml, *P*=0.038] between the two groups were statistically significant. The result may indicate that SARS-CoV-2 infection affects pituitary and thyroid function differently.

**Table 3:** Comparison of thyroid function between Normonatremia group and hyponatremia group.

	Normonatremia (*n*=50)	Hyponatremia (*n*=54)	*P*-value
Thyroid function			
FT3 (pmol/L)	3.7 ± 0.9	3.1 ± 0.9	**0.001**
FT4 (pmol/L)	16.9 (15.2–18.8)	15.7 (13.2–21.1)	0.507
FT3/FT4	0.23 (0.18–0.25)	0.21 (0.16–0.27)	0.486
TSH (uIU/ml)	2.2 (1.2–3.4)	1.4 (0.8–2.4)	**0.038**
CT assessment			
TSS	2.3 (2.0–4.0)	2.8 (2.0–3.6)	0.172

Values are expressed as mean (± standard deviation) or median (interquartile range). FT3, free triiodothyronine; FT4, free thyroxine; TSH, thyroid stimulating hormone; TSS, total severity score.

A P-value <0.05 was considered statistically significant, shown in bold.

We performed a correlation analysis further to explore the relationship between FT3 and serum sodium. Spearman correlation analysis showed that there was a positive correlation between FT3 and serum sodium (*r_s_=0.358, P*< 0.001) (**Table 4**). The higher the level of FT3, the higher the serum sodium of patients, but this correlation was weak.

**Table 4:** Correlation between FT3 and serum sodium.

	Correlation coefficient(*r*)	Spearman *P*-value	95% CI	
			Low	High
FT3-Serum sodium	0.358	<0.001	0.172	0.519

CI, confidence intervals; FT3, free triiodothyronine.

## 4 Discussion

Hyponatremia is a common electrolyte disorder associated with high morbidity and mortality, about 30% in hospitalized patients, and the incidence is higher in intensive care units (17). Compared to patients with pneumonia, COVID-19 patients with a significantly higher risk of hyponatremia (3, 18). Therefore, active prevention and treatment of hyponatremia greatly help the prognosis of the disease. Frontera and colleagues found that among patients with COVID-19, moderate (Na 121–129 mEq/L) and severe (Na ≤ 120mEq/L) hyponatremia accounted for 7% and 1% of the study population, respectively (19). In our study, which included only patients with COVID-19, the incidence of hyponatremia was 43.4%. The prevalence of mild, moderate, and severe hyponatremia was 23.3%, 7.3%, and 12.8%, respectively. The higher incidence of hyponatremia may be related to the advanced age of patients and more comorbidities, and these factors are often associated with poor prognosis. Secondly, because our data came from a large tertiary general hospital, there were more critically ill patients, so the incidence of hyponatremia in our study was high.

In this study, older age, vomiting, increased neutrophil count, and higher TSS score were associated with a higher risk of hyponatremia in COVID-19 patients. Among patients with SARS-CoV-2 infection, older age, and more coexisting conditions are associated with more severe disease, and these same factors are present in patients with hyponatremia. The study by Muhammad Anees et al. found that an elevated NLR was a risk factor for hyponatremia in hospitalized patients with COVID-19 (20). Although NLR was not proven to be a risk factor for the development of hyponatremia in our study by multivariate logistic regression, neutrophil count was proved to be a risk factor for the development of hyponatremia in COVID-19 patients by univariate or multivariate logistic regression.

The increase in platelet count can reduce the risk of hyponatremia, but the reduction effect is weak. Thrombocytopenia is another feature of SARS-CoV-2 infection, and in a retrospective study of 1,476 hospitalized COVID-19 patients, 20.7% were found to have thrombocytopenia, and thrombocytopenia was associated with increased mortality (21). We found that thrombocytopenia was more in the hyponatremia group, and the difference was statistically significant compared with the normonatremia group. The causes of thrombocytopenia were related to the direct effect of the virus on bone marrow cells and the formation of autoantibodies by platelets and their participation in immune regulation (22, 23).

ADH is generally produced by the supraoptic and paraventricular hypothalamic nuclei, stored and released from the posterior pituitary. ADH can also be derived from non-pituitary sources, and excessive release of the hormone from other sites results in SIAD. SIAD can be induced by various factors, including tumors, infections such as pneumonia and meningitis, and neurological diseases such as stroke (24). The effect of SIAD on hyponatremia in community-acquired pneumonia has been confirmed by studies (25), and SIAD is considered the leading cause of hyponatremia in COVID-19 patients. IL-6 is one of the most critical cytokines in inflammatory syndrome, causing pathological changes after SARS-CoV-2 infection (26). Elevated IL-6 levels can induce ADH secretion by directly stimulating the hypothalamus and inducing alveolar basement membrane damage and pulmonary hypoxia (27). Second, after SARS-CoV-2 infection, activated immune cells (mainly T and B lymphocytes) and released proinflammatory cytokines stimulate immune cells to release stored ADH (28).

In the study by A Berni et al., IL-6 was elevated in 17 of 29 patients with SARS-CoV-2 infection and inversely correlated with serum sodium concentration (29). In our study, IL-6 was also elevated in the hyponatremia group, and the difference was statistically significant compared with the normonatremia group. Furthermore, in the linear analysis, we also found a weak negative correlation between IL-6 and serum sodium (**Supplementary Tables S3**, **S4**). This result of our study may further support the idea of a nonosmotic release of ADH associated with IL-6.

With the rapid spread of COVID-19 worldwide, many scoring systems for lung assessment have been released. Chest CT severity score, total severity score, modified total severity score, and other scoring methods have excellent reliability in clinical assessment (11, 30). Peijie Lyu and colleagues found that qualitative or quantitative chest CT measures can assess the clinical severity of COVID-19 pneumonia (31). Miklos Szabo et al. found that the chest CT scoring system (CCTS) and specific chest CT patterns can predict ventilation requirements and mortality in COVID-19 (32). In this study, we used the TSS semi-quantitative method to assess the severity of lung lesions in patients with COVID-19 and then correlate it with hyponatremia. Because our study included a small number of patients with severe or critical lung illness, we modified the CT score of the lung to make it easier to identify mild and moderate pulmonary infections and to explore their effect on hyponatremia. The results showed that there was a significant difference in total severity score between the normonatremia group and the hyponatremia group, which suggested that CT score may be a risk factor for hyponatremia, and the results of multivariate logistic regression also proved this, the higher the TSS score, the higher the risk of hyponatremia.

CT score can predict the severity of pneumonia after SARS-CoV-2 infection (31–33), and hyponatremia can be caused by SARS-CoV-2 infection (34, 35). Many studies have confirmed these conclusions. To the best of our knowledge, this study is the first to correlate CT score with hyponatremia, and further exploration showed a weak inverse association between TSS and serum sodium (**Supplementary Table S5**), suggesting that not only can CT score predict the risk of hyponatremia, but it also seems to predict the severity of hyponatremia. Of course, we still need to do much validation. Given the widespread and convenient use of chest CT examination in clinical practice, our results are encouraging, which means that CT score can not only predict the occurrence of hyponatremia after SARS-CoV-2 infection but also provide new ideas for evaluating the association between other lung infections and hyponatremia. Although derived from inpatients, our findings may also be helpful in the outpatient setting since chest CT is routinely performed based on lung lesions. CT scores can predict the development of hyponatremia before serologic tests, which may facilitate early intervention in the outpatient setting.Multiple studies have reported impaired thyroid function in COVID-19 patients, including decreased TSH and T3 levels, decreased TSH levels alone, decreased TSH and increased T4 levels, and decreased TSH and FT4 (36–39). The causes of thyroid dysfunction may be related to a direct effect of COVID-19 on thyroid follicular cells or to disturbances in immune function (40, 41). Our study found that TSH and FT3 in patients with hyponatremia were lower than those with normal serum sodium. Similarly, W Gao et al. found that FT3 concentration was significantly lower in patients with severe COVID-19 than in non-severe patients, and FT3 reduction could be used as an independent predictor of all-cause mortality in patients with severe COVID-19 (42). We considered that the reasons for the lower TSH and FT3 in the hyponatremia group were as follows (1): After SARS-CoV-2 infection, the pituitary cells of patients were damaged (43), resulting in the increased release of ADH and the decreased secretion of TSH. The increased release of ADH can cause dilute hyponatremia, while the decreased secretion of TSH can cause a decrease in FT4 and FT3 (2). Cytokine IL-6 is involved in SARS-CoV-2-related cytokine storm (44). Elevated IL-6 can cause the non-osmotic release of ADH and increase the occurrence of hyponatremia. McGonagle et al. found that increased IL-6 and TNF-α were associated with decreased FT3 levels in patients with severe COVID-19 (45). In patients with SARS-CoV-2 infection, elevated IL-6 is associated with subacute thyroiditis, Graves’ disease, and Hashimoto’s thyroiditis (46), while abnormalities of the hypothalamic-pituitary-thyroid axis can cause a series of changes in TSH and thyroid hormones. Hyponatremia and low FT3 together affect the severity and prognosis of the disease (3). The patients in our hyponatremia group had a poorer general condition (**Supplementary Table S6**), were at higher risk for multiorgan dysfunction, and were more likely to be treated with glucocorticoids according to guideline recommendations. In contrast, the administration of glucocorticoids decreases TSH levels and inhibits the conversion of T4 to T3 while stimulating the conversion of T4 to rT3 (47, 48), the changes similar to those observed in non-thyroidal illness syndrome. Decreases in TSH and T3 are common, and the degree of decrease in T3 correlates with disease severity. Although the results of these studies were based on patients with SARS-CoV-2 infection, it also suggests that we should be aware of thyroid abnormalities in other lung lesions.

Our study also has several additional limitations: First, we did not observe the dynamic evolution of CT scores and hyponatremia in this cross-sectional study, and the lack of a certain follow-up period may make our conclusions partial. Second, the semi-quantitative CT score used in this study is subject to error and unvalidated, as well as the lack of specific serologic measures of specificity (e.g., ADH), which could attenuate the association between CT score and hyponatremia. Third, this study lacked a study of patients with hypernatremia because hypernatremia may be associated with worse outcomes (ICU admission, intubation, death). Finally, there were no statistics on vaccination status, such as the occurrence of autoimmune thyroid disease after COVID-19 vaccination, in some studies, so it is difficult to rule out the effect of this confounding factor.

In conclusion, in this study, for the first time, the semi-quantitative CT visual score was associated with hyponatremia, and the endocrine factor (thyroid function) was analyzed to clarify the relationship further. It was found that the CT score level can be used to evaluate the occurrence of hyponatremia, which can achieve early detection, prediction, and intervention in clinical practice. It is helpful to reduce the occurrence of clinical complications. Although our study population was derived from patients with SARS-CoV-2 infection, it provides a new perspective for analyzing patients with other lung lesions or endocrine abnormalities.

## 5 Conclusion

In our study, CT semi-quantitative score was associated with hyponatremia for the first time, and the endocrine factor (thyroid function) was analyzed to clarify further the association, and high TSS was found to be a risk factor for hyponatremia. Although our study population was derived from patients with SARS-CoV-2 infection, it provides a new perspective for analyzing patients with other lung lesions or endocrine abnormalities. The haze brought by COVID-19 has gradually dissipated, but new variants still exist, and the research on long COVID-19 is in the early stages. We hope our research can provide a reference for disease prevention, diagnosis, and treatment.

## Data availability statement

The original contributions presented in the study are included in the article/**Supplementary Material**. Further inquiries can be directed to the corresponding authors.

## Ethics statement

The studies involving humans were approved by the Ethics Committee of the First Hospital of Shanxi Medical University. The studies were conducted in accordance with the local legislation and institutional requirements. The participants provided their written informed consent to participate in this study. Written informed consent was obtained from the individual(s) for the publication of any potentially identifiable images or data included in this article.

## Author contributions

BW: Visualization, Writing – review & editing, Writing – original draft, Investigation, Formal analysis, Data curation. RL: Formal analysis, Data curation, Writing – review & editing, Writing – original draft, Methodology, Investigation. JH: Writing – review & editing, Visualization, Formal analysis, Data curation. YQ: Writing – review & editing, Visualization, Formal analysis, Data curation. BL: Writing – review & editing, Software, Formal analysis, Data curation. HW: Writing – review & editing, Software, Formal analysis, Data curation. ZL: Writing – review & editing, Software, Formal analysis, Data curation. YZ: Writing – review & editing, Supervision, Methodology, Funding acquisition, Conceptualization. YL: Writing – review & editing, Supervision, Project administration, Methodology, Funding acquisition, Conceptualization.
