# Supplementary material for: CT semi-quantitative score used as risk factor for hyponatremia in patients with COVID-19: a cross-sectional study
Source: Front Endocrinol (Lausanne). 2024 Jun 14;15:1342204. doi: 10.3389/fendo.2024.1342204 (PMC11211362; doi:10.3389/fendo.2024.1342204)
Supplement: Supplementary file 1 [file Table_1.docx]

Supplementary Material

# Supplementary Tables

Table S1 Demographic and clinical characteristics between different degrees of hyponatremia and normonatremia groups

|  | Normonatremia  (*n*=194) | Mild Hyponatremia  (*n*=80) | Moderate Hyponatremia  (*n*=25) | Severe Hyponatremia  (*n*=44) | *P*-value |
| --- | --- | --- | --- | --- | --- |
| **Demographic characteristics** |  | | | | |
| Age (years) | 72.0(65.0-82.0) | 80.0(68.5-86.8) | 85.0(78.5-87.5) | 78.0(69.3-84.8) | **＜0.001** |
| Male, *n* (%) | 110.0(56.7) | 57.0(71.2) | 17.0(68.0) | 17.0(38.6) | **0.003** |
| Body mass index (kg/m^2^) | 23.9(21.1-26.8) | 24.3(22.9-27.0) | 23.1(20.1-27.4) | 23.0(19.7-24.8) | 0.094 |
| **Vital signs** |  |  |  |  |  |
| Body temperature (℃) | 36.5(36.3-36.8) | 36.6(36.2-37.2) | 36.6(36.2-37.0) | 36.6(36.4-36.9) | 0.324 |
| Pulse (Times/min) | 80.0(76.0-90.0) | 80.0(70.5-90.0) | 82.0(78.0-97.5) | 78.0(72.5-88.0) | 0.227 |
| SBP (mmHg) | 130.0(117.8-139.0) | 133.0(120.3-145.8) | 124.0(108.0-136.5) | 132.0(118.5-147.0) | 0.087 |
| DBP (mmHg) | 76.0(70.0-82.0) | 76.0(68.0-85.0) | 71.0(62.5-78.0) | 78.0(71.3-82.8) | 0.327 |
| **Symptoms** |  |  |  |  |  |
| Fever, *n* (%) | 28.0(14.4) | 20.0(25.0) | 4.0(16.0) | 7.0(15.9) | 0.235 |
| Shortness of breath, *n* (%) | 93.0(47.9) | 40.0(50.0) | 10.0(40.0) | 10.0(22.7) | **0.014** |
| Cough/ Expectoration, *n* (%) | 153.0(78.9) | 61.0(76.3) | 19.0(76.0) | 24.0(54.5) | **0.010** |
| Muscle soreness, *n* (%) | 26.0(13.4) | 9.0(11.3) | 2.0(8.0) | 3.0(6.8) | 0.556 |
| Disturbance of consciousness, *n* (%) | 13.0(6.7) | 3.0(3.8) | 2.0(8.0) | 11.0(25.0) | **0.002** |
| Poor appetite, *n* (%) | 124.0(63.9) | 50.0(62.5) | 18.0(72.0) | 35.0(79.5) | 0.186 |
| Vomiting, *n* (%) | 11.0(5.7) | 5.0(6.3) | 2.0(8.0) | 14.0(31.8) | **<0.001** |
| Diarrhea, *n* (%) | 6.0(3.1) | 4.0(5.0) | 0 | 2.0(4.5) | 0.478 |
| **Comorbidities** |  | | | | |
| Diabetes, *n* (%) | 45.0(23.2) | 30.0(37.5) | 5.0(20.0) | 9.0(20.5) | 0.058 |
| Hypertension, *n* (%) | 97.0(50.0) | 40.0(50.0) | 10.0(40.0) | 21.0(47.7) | 0. 814 |
| Coronary heart disease, *n* (%) | 31.0(16.0) | 11.0(13.8) | 2.0(8.0) | 3.0(6.8) | 0.294 |
| Cerebral infarction, *n* (%) | 28.0(14.4) | 10.0(12.5) | 5.0(20.0) | 7.0(15.9) | 0.827 |
| Thyroid dysfunction, *n* (%) | 7.0(3.6) | 3.0(3.8) | 0 | 1.0(2.3) | 0.586 |
| Pulmonary disease, *n* (%) | 5.0(2.6) | 3.0(3.8) | 5.0(20.0) | 1.0(2.3) | **0.016** |
| **Medication at admission** |  | | | | |
| Diuretics, *n* (%) | 6.0(3.1) | 6.0(7.5) | 1.0(4.0) | 8.0(18.2) | **0.008** |
| ACEI/ ARBs, *n* (%) | 15.0(7.7) | 10.0(12.5) | 5.0(20.0) | 2.0(4.5) | 0.136 |
| Glucocorticoids, *n* (%) | 15.0(7.7) | 4.0(5.0) | 1.0(4.0) | 3.0(6.8) | 0.787 |
| **Laboratory tests** |  | | | | |
| Leukocyte (×10^9^/L) | 5.6(4.1-7.8) | 5.7(4.5-8.1) | 6.1(4.2-8.8) | 8.4(5.6-10.8) | **＜0.001** |
| Hemoglobin (g/L) | 137.0(124.0-148.0) | 136.0(122.0-148.8) | 129.0(113.5-138.5) | 135.5(124.3-144.8) | 0.163 |
| Platelets (×10^9^/L) | 194.0(142.0-246.0) | 165.5(127.3-215.0) | 175.0(103.0-236.5) | 180.0(132.8-233.8) | 0.080 |
| Lymphocytes (×10^9^/L) | 1.0(0.7-1.4) | 0.8(0.6-1.2) | 0.6(0.5-0.9) | 0.6(0.4-1.0) | **＜0.001** |
| Neutrophils (×10^9^/L) | 3.8(2.6-5.9) | 4.1(3.0-6.6) | 5.7(2.8-7.7) | 6.6(4.1-9.3) | **＜0.001** |
| NLR | 3.8(2.4-6.5) | 4.7(3.1-8.5) | 8.4(4.3-14.7) | 12.0(6.0-21.8) | **＜0.001** |
| Blood glucose (mmol/L) | 6.7(5.8-8.8) | 7.3(6.2-9.7) | 6.5(6.0-8.6) | 6.9(6.1-8.5) | 0.091 |
| ALT (U/L) | 20.5(13.0-35.3) | 26.0(17.0-43.0) | 26.0(21.0-36.5) | 28.5(16.5-37.5) | 0.137 |
| AST (U/L) | 27.0(21.0-41.0) | 35.0(23.3-53.0) | 43.0(27.0-66.5) | 40.0(25.0-59.5) | **＜0.001** |
| Albumin (g/L) | 35.8(33.0-39.3) | 34.4(32.0-37.6) | 33.6(30.4-38.0) | 35.2(30.8-39.1) | 0.147 |
| BUN (mmol/L) | 5.3(4.0-7.3) | 5.7(4.5-7.2) | 6.2(4.0-12.1) | 4.3(3.6-7.2) | **0.046** |
| SCr (μmol/L) | 67.0(57.0-81.6) | 75.5(63.2-92.4) | 72.0(61.5-95.0) | 55.5(47.0-73.0) | **＜0.001** |
| eGFR (mL/min/1.73m^2^) | 91.5(75.7-100.1) | 83.2(64.7-92.8) | 79.9(63.4-88.9) | 93.2(80.6-99.3) | **＜0.001** |
| Potassium (mmol/L) | 3.9(3.6-4.3) | 4.0(3.6-4.4) | 3.9(3.4-4.4) | 3.7(3.0-4.1) | **0.038** |
| Chlorine (mmol/L) | 102.5(100.4-104.8) | 97.6(94.9-99.5) | 93.3(90.7-94.5) | 83.4(73.3-86.8) | **＜0.001** |
| PT (s) | 13.6(12.8-14.4) | 13.7(12.8-14.3) | 13.1(12.5-13.7) | 13.3(12.6-14.4) | 0.235 |
| APTT (s) | 31.6(29.3-33.7) | 32.8(30.4-36.2) | 32.5(27.6-34.9) | 31.3(29.5-35.0) | 0.063 |
| FDP (ug/ml) | 4.7(1.8-163.5) | 4.0(1.9-134.3) | 5.6(2.3-203.0) | 4.3(1.9-75.5) | 0.643 |
| D dimer (mg/L) | 2.2(0.3-4.5) | 1.3(0.3-4.5) | 1.8(0.3-4.5) | 2.0(0.4-3.5) | 0.893 |
| PCT (ng/ml) | 0.26(0.16-0.35) | 0.29(0.21-0.41) | 0.27(0.16-0.95) | 0.34(0.22-1.14) | 0.059 |
| BNP (ng/L) | 52.6(26.8-122.8) | 90.5(41.7-280.5) | 132.0(65.7-298.0) | 115.4(67.2-223.9) | **＜0.001** |
| hs-cTnT (pg/ml) | 9.5(4.4-19.6) | 18.2(6.6-37.3) | 12.2(7.5-24.8) | 11.7(7.2-24.4) | **＜0.001** |
| **CT assessment** |  | | | | |
| TSS (scores) | 3.0(2.0-4.5) | 4.0(2.5-6.0) | 3.5(3.0-5.0) | 3.3(2.0-4.9) | **0.003** |

Values are expressed as median (interquartile range) or number (percentage). Serum creatinine (SCr) measurements were used to calculate the estimated Glomerular Filtration Rate (eGFR) by using the 2021 Chronic Kidney Disease Epidemiology Collaboration (2021 CKD-EPI) Creatinine equation. Abbreviations: SBP, systolic blood pressure; DBP, diastolic blood pressure; ACEI/ARB, angiotensin-converting enzyme inhibitor/ angiotensin receptor blocker; NLR, neutrophil to lymphocyte ratio; ALT, Alanine aminotransferase; AST, aspartate aminotransferase; BUN, blood urea nitrogen; Cr, creatinine; eGFR, estimated glomerular filtration rate; PT, prothrombin time; APTT, activated partial thromboplastin time; FDP, fibrinogen degradation products; PCT, procalcitonin; BNP, brain natriuretic peptide; hs-cTnT, high-sensitivity cardiac troponin T; TSS, total severity score.

A *P*-value <0.05 was considered statistically significant, which is shown in bold

Table S2 ICC results for Radiologists 1 and 2

|  | Intraclass Correlation | Radiologist 1 | Radiologist 2 | 95% CI | | *P*-value |
| --- | --- | --- | --- | --- | --- | --- |
|  |  |  |  | Lower Bound | Upper Bound |  |
| Average Measure | 0.953 | 3.7±2.1 | 3.6±2.1 | 0.942 | 0.962 | 0.000 |

Abbreviations: CI, confidence intervals

Table S3 Comparison of IL-6 between Normonatremia group and hyponatremia group

|  | Normonatremia  (*n*=53) | Hyponatremia  (*n*=51) | *P*-value |
| --- | --- | --- | --- |
| IL-6(pg/ml) | 23.4(7.5-60.8) | 42.2(21.5-92.3) | 0.011 |

Abbreviations: IL-6, interleukin-6.

Table S4 Correlation between IL-6 and serum sodium

|  | Correlation coefficient(r) | Spearman  *P*-value | 95% CI | |
| --- | --- | --- | --- | --- |
|  |  |  | Low | High |
| IL-6 - Serum sodium | -0.294 | 0.002 | -0.465 | -0.102 |

Abbreviations: CI, confidence intervals; IL-6, interleukin-6.

Table S5 Correlation between TSS and serum sodium

|  | Correlation coefficient(r) | Spearman  P-value | 95% CI | |
| --- | --- | --- | --- | --- |
|  |  |  | Low | High |
| TSS- Serum sodium | -0.159 | 0.003 | -0.263 | -0.050 |

Abbreviations: CI, confidence intervals; TSS, total severity score.

Table S6 Demographic and clinical characteristics of subgroup analysis specific to thyroid function

|  | Normonatremia  (*n*=50) | Hyponatremia  (*n*=54) | *P*-value |
| --- | --- | --- | --- |
| **Demographic characteristics** |  | | |
| Age (years) | 71.0(61.8-79.0) | 76.0(66.0-84.2) | 0.092 |
| Male, *n* (%) | 23.0(46.0) | 25.0(46.3) | 0.976 |
| Body mass index (kg/m^2^) | 24.0(20.8-27.0) | 24.0(20.8-27.3) | 0.979 |
| **Vital signs** |  |  |  |
| Body temperature (℃) | 36.5(36.3-36.7) | 36.5(36.3-37.0) | 0.474 |
| Pulse (Times/min) | 79.5(75.8-86.5) | 78.0(72.0-95.0) | 0.896 |
| SBP (mmHg) | 131.0(116.5-139.3) | 130.5(116.8-143.5) | 0.825 |
| DBP (mmHg) | 76.0(69.0-82.3) | 76.0(68.8-84.0) | 0.951 |
| **Symptoms** |  |  |  |
| Fever, *n* (%) | 6.0(12.0) | 12.0(22.2) | 0.169 |
| Shortness of breath, *n* (%) | 22.0(44.0) | 23.0(42.6) | 0.885 |
| Cough/ Expectoration, *n* (%) | 40.0(80.0) | 36.0(66.7) | 0.126 |
| Muscle soreness, *n* (%) | 8.0(16.0) | 4.0(7.4) | 0.288 |
| Disturbance of consciousness, *n* (%) | 3.0(6.0) | 9.0(16.7) | 0.163 |
| Poor appetite, *n* (%) | 37.0(74.0) | 44.0(81.5) | 0.358 |
| Vomiting, *n* (%) | 3.0(6.0) | 13.0(24.1) | **0.023** |
| Diarrhea, *n* (%) | 2.0(4.0) | 3.0(5.6) | 1.000 |
| **Comorbidities** |  | | |
| Diabetes, *n* (%) | 13.0(26.0) | 11.0(20.4) | 0.496 |
| Hypertension, *n* (%) | 28.0(56.0) | 23.0(42.6) | 0.172 |
| Coronary heart disease, *n* (%) | 7.0(14.0) | 3.0(5.6) | 0.260 |
| Cerebral infarction, *n* (%) | 8.0(16.0) | 7.0(13.0) | 0.660 |
| Thyroid dysfunction, *n* (%) | 4.0(8.0) | 3.0(5.6) | 0.916 |
| Pulmonary disease, *n* (%) | 3.0(6.0) | 4.0(7.4) | 1.000 |
| **Medication at admission** |  | | |
| Diuretics, *n* (%) | 3.0(6.0) | 7.0(13.0) | 0.384 |
| ACEI/ ARBs, *n* (%) | 5.0(10.0) | 5.0(9.3) | 0.898 |
| Glucocorticoids, *n* (%) | 2.0(4.0) | 3.0(5.6) | 1.000 |
| **Laboratory tests** |  | | |
| Leukocyte (×10^9^/L) | 5.4(4.4-8.3) | 5.9(4.8-9.9) | 0.234 |
| Hemoglobin (g/L) | 135.0(123.8-148.3) | 133.0(123.0-146.5) | 0.494 |
| Platelets (×10^9^/L) | 200.5(142.0-280.8) | 157.7(117.3-223.5) | **0.013** |
| Lymphocytes (×10^9^/L) | 1.2(0.7-1.7) | 0.8(0.5-1.2) | **0.001** |
| Neutrophils (×10^9^/L) | 3.8(2.7-7.0) | 4.5(3.0-8.6) | 0.164 |
| NLR | 3.5(2.4-5.7) | 7.1(3.3-10.8) | **0.003** |
| Blood glucose (mmol/L) | 6.2(5.3-8.1) | 7.0(6.0-8.9) | 0.148 |
| ALT (U/L) | 22.0(13.0-34.0) | 30.5(21.5-44.3) | **0.015** |
| AST (U/L) | 27.0(22.0-40.0) | 45.0(30.1-68.5) | **＜0.001** |
| Albumin (g/L) | 37.5(35.0-40.3) | 35.1(33.0-38.9) | **0.029** |
| BUN (mmol/L) | 4.9(3.7-7.0) | 4.8(3.8-8.1) | 0.733 |
| SCr (μmol/L) | 65.3(56.3-80.6) | 71.6(51.5-84.6) | 0.782 |
| eGFR (mL/min/1.73m^2^) | 93.7(75.5-102.4) | 85.1(69.2-98.6) | 0.173 |
| Potassium (mmol/L) | 4.0(3.5-4.3) | 3.9(3.4-4.2) | 0.260 |
| Chlorine (mmol/L) | 102.8(101.0-104.6) | 91.3(85.3-99.2) | **＜0.001** |
| PT (s) | 13.3(12.7-14.4) | 13.4(12.6-14.3) | 0.920 |
| APTT (s) | 31.6(30.2-33.5) | 32.1(29.9-37.3) | 0.517 |
| FDP (ug/ml) | 3.2(1.5-6.0) | 3.6(1.4-10.7) | 0.755 |
| D dimer (mg/L) | 0.6 (0.2-3.7) | 0.7(0.2-3.6) | 0.935 |
| PCT (ng/ml) | 0.2(0.1-0.3) | 0.3(0.2-0.5) | **0.037** |
| BNP (ng/L) | 58.3(20.8-135.1) | 99.5(46.1-366.4) | **0.006** |
| hs-cTnT (pg/ml) | 9.4(3.7-19.7) | 10.9(6.0-27.2) | 0.163 |

Values are expressed as mean (± standard deviation), median (interquartile range), or number (percentage). Serum creatinine (SCr) measurements were used to calculate the estimated Glomerular Filtration Rate (eGFR) by using the 2021 Chronic Kidney Disease Epidemiology Collaboration (2021 CKD-EPI) Creatinine equation. Abbreviations: SBP, systolic blood pressure; DBP, diastolic blood pressure; ACEI/ARB, angiotensin-converting enzyme inhibitor/ angiotensin receptor blocker; NLR, neutrophil to lymphocyte ratio; ALT, Alanine aminotransferase; AST, aspartate aminotransferase; BUN, blood urea nitrogen; Cr, creatinine; eGFR, estimated glomerular filtration rate; PT, prothrombin time; APTT, activated partial thromboplastin time; FDP, fibrinogen degradation products; PCT, procalcitonin; BNP, brain natriuretic peptide; hs-cTnT, high-sensitivity cardiac troponin T.

A *P*-value <0.05 was considered statistically significant, which is shown in bold
